# Motorcycle helmet use to reduce road traffic deaths in Thailand

**DOI:** 10.2471/BLT.18.215509

**Published:** 2018-08-01

**Authors:** Akihiro Nishi, Phathai Singkham, Yohsuke Takasaki, Masao Ichikawa, Witaya Chadbunchachai, Kenji Shibuya, Chanuantong Tanasugarn

**Affiliations:** a Department of Epidemiology, University of California, Los Angeles Fielding School of Public Health, 650 Charles E Young Dr S, Los Angeles, California 90095, United States of America.; b Bureau of Noncommunicable Diseases, Ministry of Public Health, Nonthaburi, Thailand.; c The Partnership Project for Global Health and Universal Health Coverage, Japan International Cooperation Agency, Bangkok, Thailand.; d Faculty of Medicine, University of Tsukuba, Tsukuba, Japan.; e World Health Organization Collaborating Centre for Injury Prevention and Safety Promotion, Khon Kaen Regional Hospital, Khon Kaen, Thailand.; f Institute of Global Health Policy Research, National Center for Global Health and Medicine, Tokyo, Japan.; g Department of Health Education and Behavioral Sciences, Mahidol University, Bangkok, Thailand.

Thailand faces road safety challenges. The country has a high rate of road traffic deaths, with 36.2 deaths per 100  000 population per year according to the World Health Organization (WHO) estimate in 2012,^1^ which is the highest road death rate in South-East Asia. Reflecting the Sustainable Development Goal 3.6 of halving the number of global deaths and injuries from road traffic crashes by 2020, the national action plan *Decade for action for road safety (2011–2020)*^2^ aims to reduce the road traffic death rate to less than 10 deaths per 100  000 population per year by 2020. However, achieving this goal seems challenging in Thailand, because the issues that need to be addressed are multilayered and relate to individual, social and environmental factors.

Increasing motorcycle helmet use in Thailand could help achieve this injury reduction target, since motorcycles are the most popular private transportation vehicle for most households, especially in remote areas where there is no official public transportation system. Motorcycle drivers and/or passengers account for 73% of road traffic deaths,^1^ compared with 15% in the United States of America.^3^ Reducing traffic deaths of motorcycle users, who are vulnerable road users, is a top priority for the Thai government.

Motorcycle helmet use saves lives. A review shows that the adequate use of a certified helmet on roads can decrease the mortality risk by 40% and the risk of head injury by 70%.^4^ In Thailand, a universal motorcycle helmet use law (for drivers since 1996 and for passengers since 2007) has led to a substantial increase in the proportion of motorcycle helmet use, from 5% in 1994 to 23% in 1997.^5^ However, the pace of further increase in motorcycle helmet use has been low: one recent study reported that interventions, such as awareness raising campaigns, improved the proportion of helmet use, but the milestone of 50% nationwide has not been achieved.^6^^,^^7^ A mixed intervention model, relevant both to people’s needs and community contexts, is needed.

One good sign is that the proportion of helmet use in Bangkok is now above 80%.^8^ Although motorcycle drivers may think they will not be involved in traffic crashes and that wearing a helmet is not necessary, behaviours and social norms have gradually changed. Helmet use exhibits geographic-based and age-based disparities: for example, the estimated proportion of helmet use among teenagers in Khon Kaen is 14%.^8^ Teenagers living in remote areas can be one of the prioritized targets for future intervention studies.

We estimate that an increase in the proportion of helmet use from 44%, the nationwide figure in 2010^8^, to 90% would lead to a 23% immediate reduction of the total road traffic deaths among motorcycle users. We assume that the chances of being involved in a traffic accident are the same for helmet and non-helmet users and that if non-helmet users wore one, 40% of the potential deaths could be prevented.^4^

In response to the *Decade of action for road safety (2011–2020)*, the National Road Safety Committee, Royal Thai Police, Ministries of Transportation, Public Health, Education and Labour, National Institute for Emergency Medicine, Thai Health Promotion Foundation, ThaiRoads Foundation, Road Safety Policy Foundation and other agencies are focusing efforts on road safety good practices. Measures taken include enhancing road surveillance and emergency care systems and securing financial resources for road safety. However, national and international researchers need to be more engaged in the country’s challenge to better respond to the Phuket commitment at the WHO South-East Asia Ministerial Meeting on Accelerating Actions for implementation of *Decade of action for road safety (2011–2020)*.^9^ In particular, there should be more researchers in the fields of medical and social sciences who work on the promotion of motorcycle helmet use in Thailand. Current research on the impact of helmet use on motorcycle-related injuries is insufficient. For example, as of December 2017, a PubMed search using “Thailand” and “helmet” as keywords, for the period between 2008 and 2017, only produced an average of 1.4 published papers per year. The upcoming Safety 2018 World Conference^10^ will be a good opportunity to recruit more researchers and discuss the role of motorcycle helmet use in implementing Thailand’s national action plan.
